# MicroRNA-532-3p regulates mitochondrial fission through targeting apoptosis repressor with caspase recruitment domain in doxorubicin cardiotoxicity

**DOI:** 10.1038/cddis.2015.41

**Published:** 2015-03-12

**Authors:** J-X Wang, X-J Zhang, C Feng, T Sun, K Wang, Y Wang, L-Y Zhou, P-F Li

**Affiliations:** 1 National Key Laboratory of Biomembrane and Membrane Biotechnology, Institute of Zoology, Chinese Academy of Sciences, Beijing, China; 2 Institute for Translational Medicine, College of Medicine, Qingdao University, Qingdao, China; 3 College of Medicine, University of Illinois at Chicago, Chicago, IL, USA

## Abstract

Doxorubicin (DOX) is a wide-spectrum antitumor drug, but its clinical application is limited by its cardiotoxicity. However, the mechanisms underlying DOX-induced cardiomyopathy remain mostly unclear. Here we observed that apoptosis repressor with caspase recruitment domain (ARC) was downregulated in mouse heart and cardiomyocytes upon DOX treatment. Furthermore, enforced expression of ARC attenuated DOX-induced cardiomyocyte mitochondrial fission and apoptosis. ARC transgenic mice demonstrated reduced cardiotoxicity upon DOX administration. DOX-induced mitochondrial fission required the activity of dynamin-related protein 1 (Drp1). In elucidating the molecular mechanism by which ARC was downregulated upon DOX treatment, miR-532-3p was found to directly target ARC and participated in DOX-induced mitochondrial fission and apoptosis. MiR-532-3p was not involved in DOX-induced apoptosis in cancer cells. Taken together, these findings provide novel evidence that miR-532-3p and ARC constitute an antiapoptotic pathway that regulates DOX cardiotoxicity. Therefore, the development of new therapeutic strategies based on ARC and miR-532-3p is promising for overcoming the cardiotoxicity of chemotherapy for cancer therapy.

Doxorubicin (DOX) is one of the most widely used anticancer drugs. A large number of patients treated with DOX develop carditoxicity, which can lead to congestive heart failure.^1, 2^ The exact mechanisms of DOX cardiotoxicity remain unclear. Multiple mechanisms by which DOX-induced irreversible myocaridial injures have been proposed, including reactive oxygen species (ROS) production, lipid peroxidation, mitochondrial impairment, intracellular calcium dysregulation and direct suppression of heart-special gene expression.^3, 4, 5^ Most of these mechanisms ultimately result in the activation of apoptotic signaling, leading to progressive loss of cardiac myocytes.^2^ Considering the limited capacity for proliferation, it is essential to understand the molecular signaling underlying DOX-induced cardiomyocyte death in order to establish interventions that can effectively prevent cardiac cell loss and, thereby, preserve cardiac function.

The heart has evolutionarily developed a highly expressed antiapoptotic protein, apoptosis repressor with caspase recruitment domain (ARC).^6^ ARC was initially discovered as an endogenous apoptosis inhibitor in postmitotic cells, such as cardiomyocytes, skeletal muscle cells and neurons. It can antagonize both intrinsic and extrinsic apoptosis signaling pathways.^6, 7, 8^ ARC has a role in maintaining physiological cardiac function. Under pathological conditions, ARC is downregulated, which may contribute to the occurrence of many heart diseases, such as hypertrophy, heart failure and cardiac infraction.^9, 10^ ARC-deficient mice exerted significantly accelerated cardiomyopathy under conditions of cardiac ischemia or pressure overload.^11^ Our previous work showed heart of cardiac-special ARC transgenic mice were more resistant to ischemia injury and hypertrophic stimuli.^12^ As for DOX cardiotoxicity, it was reported that ARC was downregulated in cardiomyocytes exposed to DOX and led to a significant induction of apoptosis.^5^ Enforced expression of ARC dramatically increased the resistance of cardiomyocytes to undergo apoptotic cell death following DOX administration.^5^ But the mechanism by which ARC was downregulated during this process is largely unknown. Our previous work has also proved that highly expressed ARC contributed to cancer cell resistance to chemotherapy by targeting the mitochondrial fission machinery.^13^ However, whether ARC inhibits mitochondrial fission in cardiomyocytes upon DOX treatment remains to be investigated.

MicroRNAs (miRNAs) are a class of small non-coding RNAs and negative regulators of target genes by altering mRNA translation or stability.^14^ MiRNAs functionally participated in a wide variety of physiological or pathological processes.^15^ MiRNAs can regulate cardiac function such as the conductance of electrical signal, heart muscle contraction, heart growth and morphogenesis.^16^ Manipulation of miRNA can be developed to therapeutic approaches. However, it is not yet clear whether miRNAs are involved in the regulation of DOX cardiotoxicity. In our previous study, we have demonstrated that miR-185 negatively regulated ARC in gastric cancer cells.^17^ However, whether miRNAs can regulate ARC in DOX cardiotoxicity is unknown.

In this study, we focused on the function of ARC in DOX cardiotoxicity both in cardiomyocytes and mice hearts. We found that ARC was downregulated in cardiomyocytes administered DOX. Enforced expression of ARC inhibited DOX-induced mitochondrial fission and apoptosis in cardiomyocytes. Heart-special ARC transgenic mice exhibited reduced cardiotoxicity. Besides, miR-532-3p could sensitize cardiomyocytes to DOX-induced mitochondrial fission and apoptosis through negatively regulating ARC expression. Our results identified a novel regulatory pathway for apoptosis involving miR-532-3p–ARC and possibly provide a valuable insight to protect against DOX cardiotoxicity during cancer chemical therapy.

## Results

### ARC regulates mitochondrial fission and apoptosis in DOX cardiotoxicity *in vitro*

**Figure 1:**
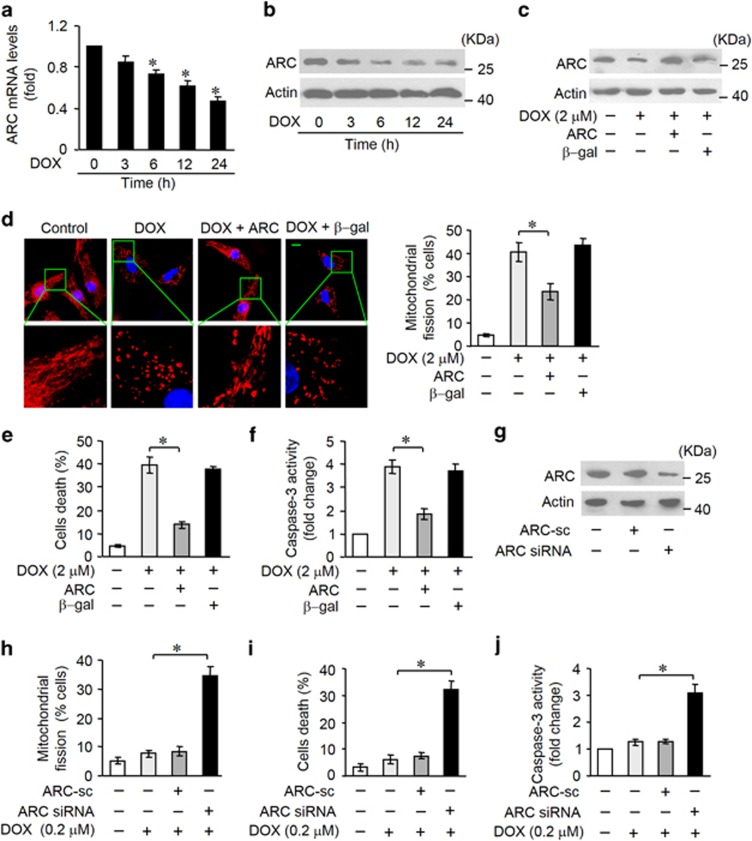
ARC is involved in DOX-induced mitochondrial fission and apoptosis in cardiomyocytes. (**a** and **b**) ARC mRNA levels (**a**) and protein levels (**b**) in neonatal rat cardiomyocytes treated with 2 *μ*M DOX at the indicated time. *P<0.05 *versus* control (untreated). (**c**–**f**) Enfored expression of ARC by infecting adenoviral ARC (**c**) attenuated DOX (2 *μ*M)-induced mitochondrial fission (**d**), cell death (**e**) and caspase-3 activation (**f**). Representative photos showed mitochondrial fission (**d**, left). Blue, DAPI (4,6-diamidino-2-phenylindole)-stained nuclei; red, MitoTracker Red CMXRos-stained mitochondria. Scale bar, 10 *μ*m. The percentage of cells undergoing mitochondrial fission were counted (**d**, right). (**g**–**j**) Mitochondrial fission (**h**), cell death (**i**) and caspase-3 activation (**j**) were increased in response to low-dose DOX (0.2 *μ*M) upon knockdown of endogenous ARC using its small interfering RNA (siRNA) (**g**). Data are expressed as the mean±S.D., *n*=3. *P<0.05

ARC is downregulated in cardiomyocytes during apoptotic stress or in the heart under pathological conditions. To study the role of ARC in DOX cardotoxicity, we treated cardiomyocytes with DOX. The expression levels of ARC were downregulated after DOX treatment at both mRNA (Figure 1a) and protein (Figure 1b) levels. Mitochondrial fission is related to initiation of apoptosis. Administration of DOX at a high dose (2 *μ*M) could induce mitochondrial fission (Supplementary Figure S1a) and cell death (Supplementary Figure S1b) in cardiomyocytes in a time-dependent manner. In addition, caspase-3 activation was increased in cardiomyocytes exposed to DOX (Supplementary Figure S1c). These data suggest that ARC can be a target of DOX in its cardiotoxicity program. Next, we detected whether ARC was involved in the regulation of DOX-induced mitochondrial fission and apoptosis. To this end, we produced a construct encoding ARC. Enforced expression of ARC was confirmed by western blotting (Figure 1c). DOX-induced mitochondrial fission (Figure 1d), cell death (Figure 1e) and caspase-3 activation (Figure 1f) were attenuated by exogenous ARC. To further confirm that it was apoptotic cell death, FACS analysis (Annexin V/ PI staining) was performed (Supplementary Figure S1d). Then we attempted to investigate the influence of endogenous ARC on cardiomyocyte susceptibility to DOX. ARC siRNA adenoviruses were constructed, and they efficiently reduced the expression levels of ARC (Figure 1g). Following low-dose DOX (0.2 *μ*M) treatment in cardiomyocytes, a limited amount of cells undergoing mitochondrial fission and death were observed. In contrast, mitochondrial fission (Figure 1h), cell death (Figure 1i and Supplementary Figure S1e) and caspase-3 activation (Figure 1j) were significantly increased in response to the same dose of DOX upon ARC knockdown. Taken together, these results suggest that ARC is able to inhibit DOX-induced mitochondrial fission and apoptosis in cardiomyocytes.

Mitochondrial fission requires the activity of dynamin-related protein 1 (Drp1).^18, 19^ Our previous work showed that ARC could prevent Drp1 accumulation in mitochondria.^13^ It is not yet clear, however, whether Drp1 influence the regulation of DOX cardiotoxicity by inducing mitochondrial fission. We generated siRNA construct for Drp1 that was able to reduce Drp1 expression levels (Supplementary Figure S2a). Knockdown of Drp1 reduced mitochondrial fragmentation and cell death upon DOX treatment (Supplementary Figures S2b and c). Enforced expression of Drp1 sensitized DOX to induce mitochondrial fission and cell death (Supplementary Figures S2d and f). DOX can induce apoptosis by initiating a mitochondrial pathway. Previous studies showed that mitochondrial fission was associated with cytochrome *C* (Cyt *C*) release and loss of mitochondrial membrane potential (*ΔΨ*m).^18, 19, 20, 21^ Knockdown of Drp1 attenuated Cyt *C* release (Supplementary Figure S2g) and loss of *ΔΨ*m (Supplementary Figure S2h) in DOX-treated cardiomyocytes. Taken together, these results suggest that Drp1-mediated mitochondrial fission is involved in the initiation of DOX cardiotoxicity.

### ARC regulates mitochondrial fission and apoptosis in DOX cardiotoxicity *in vivo*

**Figure 2:**
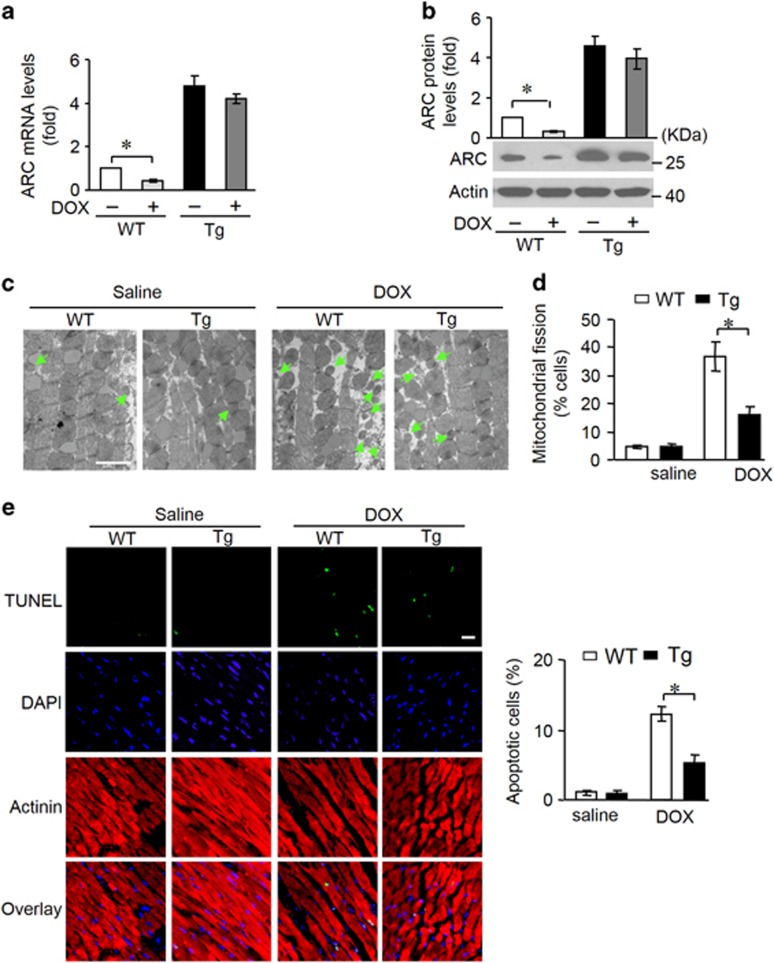
ARC regulates mitochondrial fission and apoptosis *in vivo*. (**a** and **b**) ARC mRNA levels (**a**) and protein levels (**b**) in ARC transgenic mice (Tg) and wild-type mice (WT) administered with DOX. The quantitative analysis of protein level was shown (**b**, up). *n*=3 mice per group. (**c** and **d**) Mitochondrial fission was analyzed in heart tissues of ARC Tg and WT mice administered with DOX. Scale bar, 2 *μ*m; arrows indicate fission mitochondria (**c**). Percentage of mitochondrial fission (**d**). *n*=5 mice per group. (**e**) TUNEL (terminal deoxinucleotidyl transferase-mediated dUTP-fluorescein nick-end labeling) assay was used to detect apoptotic cells in heart tissues. Green, TUNEL-positive nuclei; bule, DAPI (4,6-diamidino-2-phenylindole)-stained nuclei; red, cardiomyocytes labeled with antibody to α-actinin, scale bar, 20 *μ*m. *n*=5 mice per group. Data are presented as mean±S.D., *P<0.05

To further understand the role of ARC in DOX cardiotoxicity *in vivo*, we generated ARC transgenic mice and detected whether ARC is involved in the pathogenesis of DOX cardiotoxicity in the animal model. DOX administration induced a decrease in ARC levels in the heart tissues of mice (Supplementary Figures S3a and b). While in ARC transgenic mice, the decrease in ARC levels was attenuated upon the same condition (Figures 2a and b). By electron microscopy, mitochondrial morphology was detected (Figure 2c). Mitochondria in the hearts of saline-treated wild-type or ARC transgenic mice were similar in size; DOX administration led to a decrease in mitochondrial size in both wild-type and transgenic mice, but this effect was attenuated in transgenic mice. Mitochondria undergone fission was counted and less mitochondrial fission was observed in ARC transgenic mice upon DOX administration (Figure 2d). Apoptosis was also detected by TUNEL, upon DOX administration, the hearts of ARC transgenic mice showed less apoptosis (Figure 2e).

### ARC attenuates DOX cardiotoxicity in mice

**Figure 3:**
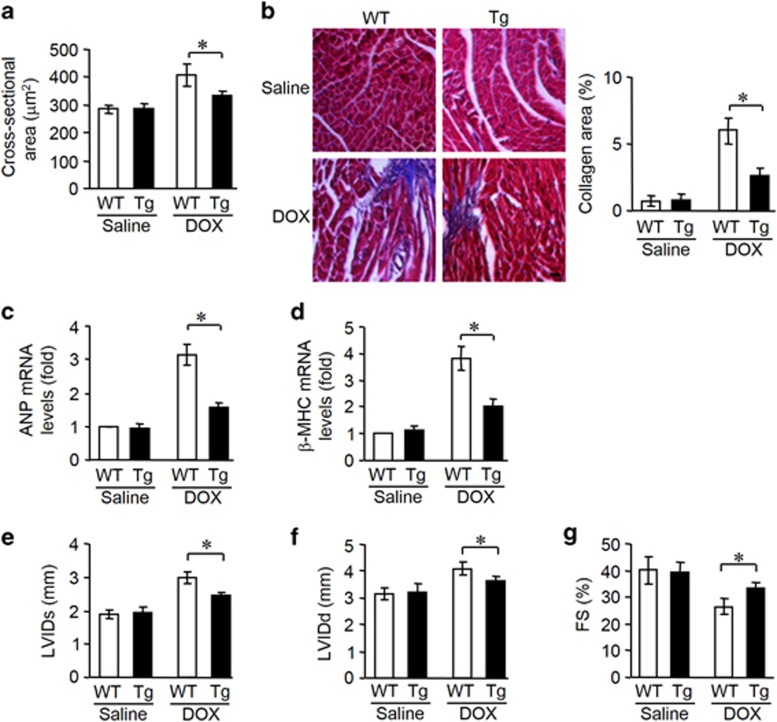
ARC attenuates DOX cardiotoxicity in mice. (**a**–**d**) ARC Tg mice are resistant to LV remodeling after DOX treatment. (**a**) Cross-sectional areas were analyzed by TRITC-conjugated wheat agglutinin staining. (**b**) The myocardial fibrosis was determined by Masson trichrome staining. Scale bar, 20 *μ*M. (**c** and **d**) The expression levels of ANP and *β*-myosin heavy chain were detected by qRT-PCR. (**e**–**g**) ARC Tg or WT mice exposed to DOX or saline as described in panel (**a**), and echocardiography was used to test heart function. LVIDs, systolic left ventricular internal diameters; LVIDd, diastolic left ventricular internal diameters; FS, fractional shortening of left ventricular diameter. Data are presented as mean±S.D., *n*=8 mice per group. *P<0.05

DOX cardiotoxicity promotes deleterious remodeling of the myocardium. Subsequently, we tested whether ARC affects the cardiac remodeling and cardiac function. ARC transgenic mice showed improved cardiac remodeling, as assessed by myocardial cross-sectional area (Figure 3a) and collagen content (Figure 3b). Changes in cardiac hypertrophic markers atrial natriuretic factor (Figure 3c) and *β*-myosin heavy chain (Figure 3d) expression profile were also attenuated in the transgenic mice. Concomitantly, the heart function was ameliorated in ARC transgenic mice as assessed by echocardiography (Figures 3e and g).

### MiR-532-3p participates in the regulation of ARC expression

**Figure 4:**
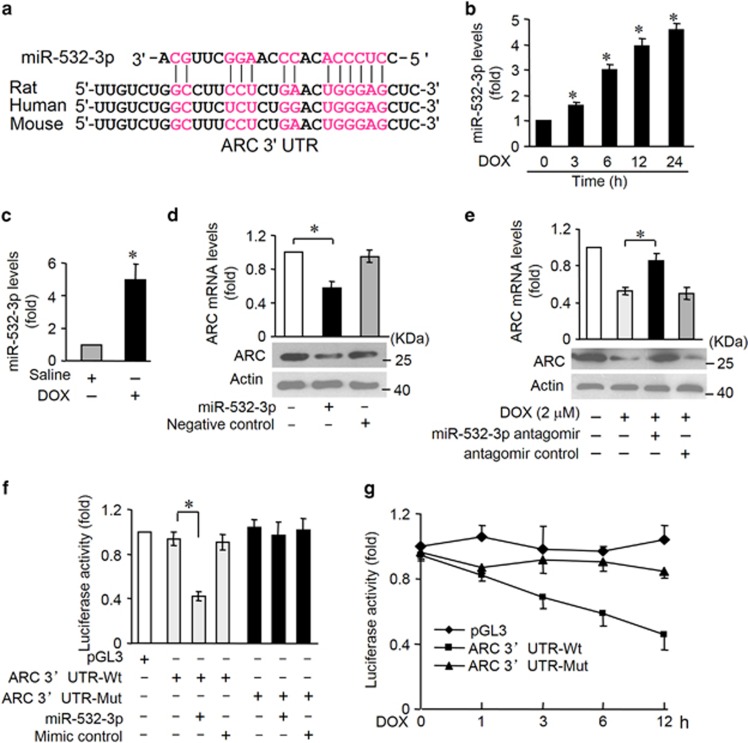
MiR-532-3p participates in the regulation of ARC expression. (**a**) Analysis of ARC 3′UTR potential binding site for miR-532-3p by RNAhybrid. Potential complementary residues are shown in red. (**b**) The miR-532-3p levels in neonatal rat cardiomyocytes treated with 2 *μ*M DOX at the indicated time. *P<0.05 *versus* control (untreated). (**c**) MiR-532-3p levels in mice administered with DOX or saline as described in Materials and Methods. *n*=5 mice per group. (**d**) ARC mRNA and protein levels in neonatal rat cardiomyocytes overexpressing miR-532-3p by transfecting with miR-532-3p mimics. (**e**) Kncokdown of endogenous miR-532-3p by transfecting its antagomirs attenuated decrease of ARC mRNA and protein levels upon DOX (2 *μ*M) for 12 h in cardiomyocytes. (**f**) Luciferase activity detected in HEK-293 transfected with synthesized miR-532-3p mimic or mimic control, along with luciferase reporter constructs as indicated. (**g**) Luciferase activity of luciferase construct ARC 3′UTR-Wt is decreased upon DOX treatment in cardiomyocytes. Data are expressed as the mean±S.D., *n*=3 except in panel (**c**). *P<0.05

MiRNA is able to suppress gene expression. To explore the underlying mechanism by which ARC is downregulated upon DOX, we tested whether miRNA can regulate ARC expression. We analyzed the 3′UTR of ARC using the RNAhybrid program and found some potential miRNAs. To explore which miRNA is involved in the regulation of ARC, we used quantitative real-time PCR (qRT-PCR) to detect miRNAs levels in response to DOX. Among several miRNAs, miR-532-3p was substantially increased (Supplementary Figure S4a). Accordingly, we focused on miR-532-3p. The potential miR-532-3p-binding site in ARC 3′UTR was shown in Figure 4a. The binding site was conserved among human, mouse and rat. The expression levels of miR-532-3p were increased in DOX-treated cardiomyocytes (Figure 4b) and in mice hearts (Figure 4c) exposed to DOX. First, we attempted to evaluate whether miR-532-3p modulated ARC expression. Enforced expression of miR-532-3p was confirmed by qRT-PCR (Supplementary Figure S4b). Overexpression of miR-532-3p led to an obvious reduction of ARC mRNA and protein levels in cardiomyocytes (Figure 4d). MiR-532-3p levels were reduced by its specific antagomir (Supplementary Figure S4c). In contrast, administration of the miR-532-3p antagomir could attenuate the decrease of ARC levels in response to DOX treatment (Figure 4e). Therefore, it seems that miR-532-3p modulates ARC expression by altering mRNA stability.

Further, we used the luciferase assay system to test whether miR-532-3p can influence the expression of ARC by directly targeting ARC 3′UTR (Supplementary Figure S4d). We cloned ARC 3′UTR containing miR-532-3p-binding site downstream of the luciferase reporter gene (ARC 3′UTR-Wt) to examine luciferase activity driven by the 3′UTR of ARC. Besides, we generated a mutated luciferase construct, ARC 3′UTR-Mut, and mutations were introduced into the miR-532-3p-binding site of ARC 3′UTR. As shown in Figure 4f, the wild-type 3′UTR of ARC exhibited a low luciferase activity in the presence of miR-532-3p, whereas the mutated 3′UTR did not show a significant response to miR-532-3p. We further investigated the effect of miR-532-3p on ARC expression in cardiomyocytes. As shown by luciferase activity, the reduction of luciferase activity driven by ARC 3′UTR upon DOX treatment was attenuated by the site-directed mutations in ARC 3′UTR (Figure 4g). In addition, we measured the miR-532-3p effect on ARC expression levels in neonatal mouse cardiomyocytes isolated from ARC transgenic mice in which ARC mRNA lacks 3′UTR. We found that overexpression of miR-532-3p had no obvious effect on ARC mRNA levels (Supplementary Figure S4e).Thus our data indicate that miR-532-3p is able to target ARC directly in DOX cardiotoxicity.

### MiR-532-3p can regulate mitochondrial fission and apoptosis in cardiomyocytes treated by DOX

**Figure 5:**
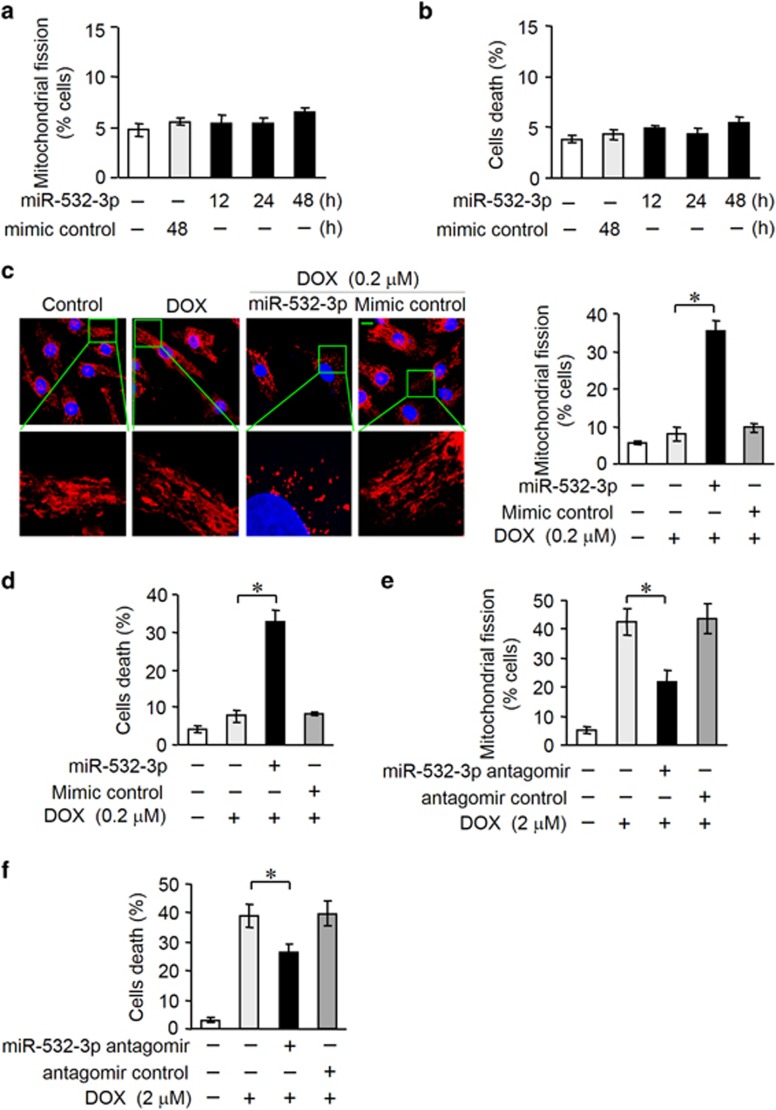
MiR-532-3p can regulate mitochondrial fission and apoptosis in cardiomyocytes treated by DOX. (**a** and **b**) Mitochondrial fission (**a**) and cell death (**b**) were not changed in neonatal rat cardiomyocytes overexpressing miR-532-3p for the indicated times. (**c** and **d**) Enfored expression of miR-532-3p enhanced mitochondrial fission (**c**) and cell death (**d**) in cardiomyocytes upon DOX (0.2 *μ*M) treatment. Representative photos showed mitochondrial fission (**c**, left). Blue, DAPI (4,6-diamidino-2-phenylindole)-stained nuclei; red, MitoTracker Red CMXRos-stained mitochondria. Scale bar, 10*μ*M. The percentage of cells undergoing mitochondrial fission were counted (**c**, right). (**e** and **f**) Inhibition of endogenous miR-532-3p using miR-532-3p antagomir prevented mitochondrial fission (**e**) and cell death (**f**) in cardiomyocytes treated with DOX (2 *μ*M). Data are expressed as the mean±S.D., *n*=3. *P<0.05

The potential role of miR-532-3p in cardiomyocytes remains largely unknown. We explored the functional role of miR-532-3p in mitochondrial fission and apoptosis in cardiomyocytes exposed to DOX. Overexpression of miR-532-3p alone had no significant effect on mitochondrial fission and apoptosis (Figures 5a and b). Then we attempted to investigate the influence of miR-532-3p on cell susceptibility to cardiotoxicity. When we enforced the expression of miR-532-3p in cardiomyocytes, the mitochondrial fission and death cells were significantly increased in response to the low-dose DOX (0.2 *μ*M) treatment (Figures 5c and d). To characterize the function of endogenous miR-532-3p in the DOX cardiotoxicity, miR-532-3p antagomir was transfected into cardiomyocytes. DOX-induced mitochondrial fission and apoptosis were attenuated by miR-532-3p antagomir (Figures 5e and f). Taken together, these results suggest that miR-532-3p regulates mitochondrial fission and apoptosis during DOX cardiotoxicity.

### MiR-532-3p regulates mitochondrial fission and apoptosis by targeting ARC

**Figure 6:**
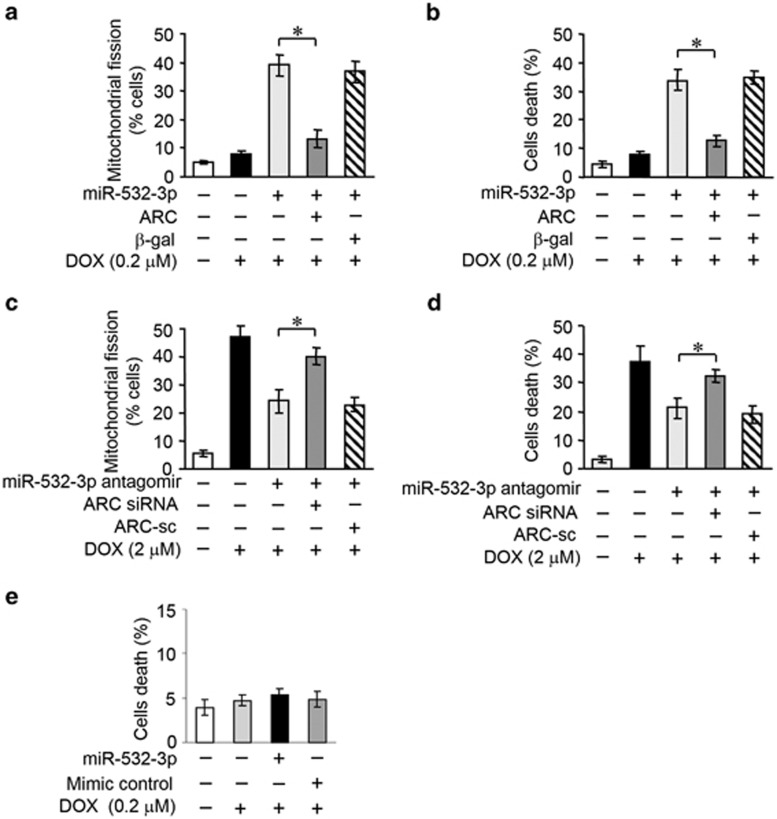
MiR-532-3p regulates mitochondrial fission and apoptosis by targeting ARC. (**a** and **b**) MiR-532-3p-increased sensitivity to DOX treatment was abolished by ARC. Mitochondrial fission (**a**) and cell death (**b**) were measured in neonatal rat cardiomyocytes upon 0.2 *μ*M DOX treatment. (**c** and **d**) MiR-532-3p antagomir-attenuated DOX treatment was abolished by endogenous ARC knockdown. Mitochondrial fission (**c**) and cell death (**d**) were measured in cardiomyocytes upon 2 *μ*M DOX treatment. (**e**) Enforced expression of miR-532-3p did not affect cell death in neonatal mouse cardiomyocytes isolated from ARC Tg mice upon DOX (0.2 *μ*M) treatment. Data are expressed as the mean±S.D., *n*=3. *P<0.05

We explored how miR-532-3p exerts its effects on the mitochondrial network. As miR-532-3p is able to suppress ARC expression, we wondered whether miR-532-3p regulated DOX sensitivity in cardiomyocytes by targeting ARC. Overexpression of miR-532-3p sensitized low-dose DOX-induced mitochondrial fission and cell death. Enforced expression of ARC showed a strong inhibitory effect on mitochondrial fission (Figure 6a) and cell death (Figure 6b) in the presence of miR-532-3p. Reduced endogenous expression level of miR-532-3p inhibited DOX-induced mitochondrial fission and cell death. Knockdown of ARC attenuated this inhibitory effect on mitochondrial fission (Figure 6c) and cell death (Figure 6d) in the presence of miR-532-3p antagomir. Then we attempted to investigate the influence of miR-532-3p on cell susceptibility to neonatal mouse cardiomyocytes isolated from ARC transgenic mice. Overexpression of miR-532-3p had no significant effect on cell death in response to the low-dose DOX (0.2 *μ*M) treatment (Figure 6e). These data suggest that miR-532-3p targets ARC in the cascades of mitochondrial fission and apoptosis during DOX cardiotoxicity.

### MiR-532-3p is not involved in DOX-induced apoptosis in cancer cells

**Figure 7:**
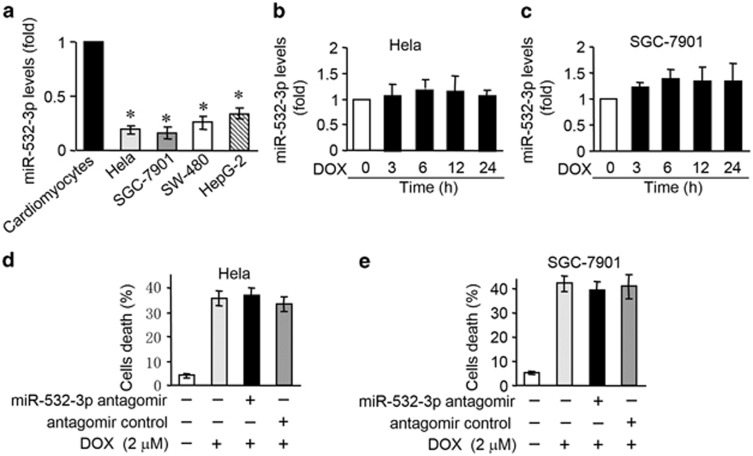
MiR-532-3p is not involved in DOX-induced apoptosis in cancer cells. (**a**) The miR-532-3p levels in cardiomyocytes, Hela, SGC-7901, SW-480 and HEPG-2 cells. *P<0.05 *versus* cardiomyocytes. (**b** and **c**) The miR-532-3p levels in Hela (**b**) and SGC-7901 (**c**) cells treated with 2 *μ*M DOX for the indicated times. (**d** and **e**) Cell death in Hela (**d**) and SGC-7901 (**e**) cells transfected with miR-532-3p antagomir and treated with 2 *μ*M DOX. Data are expressed as the mean±S.D., *n*=3

It has been previously found that ARC is also highly expressed in some malignant tumors and some type of cancer cells.^22, 23^ Our recent studies have shown that DOX led to a decrease in ARC expression levels in Hela and SGC-7901 cells.^13^ ARC contributes to cell resistance to chemotheraphy by targeting the apoptotic machinery. However, the role of miR-532-3p in DOX-induced apoptosis in cancer cells remains largely unknown. We tested whether miR-532-3p is related to apoptosis in some cancer cells exposed to DOX. MiR-532-3p was expressed at low level in cancer cells, including Hela, SGC-7901, SW-480 and HepG-2, compared with cardiomyocytes (Figure 7a). Expression levels of miR-532-3p were not changed in Hela (Figure 7b), SGC-7901 (Figure 7c), SW-480 (Supplementary Figure S5a) and HepG-2 (Supplementary Figure S5b) cells administered with DOX. Knockdown miR-532-3p had no significant effect on cell death induced by DOX in these cancer cells (Figures 7d and e and Supplementary Figures S5c and d). Taken together, it seems that miR-532-3p is not involved in DOX-induced apoptotic program in cancer cells.

## Discussion

DOX is one of the most effective chemotherapeutic agents. However, cardiotoxicity is a major challenge for effective treatment. It is quite urgent to explore potential mechanisms involved in DOX cardiotoxicity. In the present study, we have revealed for the first time that miR-532-3p-mediated suppression of ARC is a substantial causal mechanism of DOX-induced cardiac toxicity. We found that miR-532-3p was upregulated and ARC was downregulated in cardiomyocytes administered with DOX. MiR-532-3p sensitized cells to DOX-induced mitochondrial fission and apoptosis by targeting ARC. Our data may provide a new clue in understanding the molecular mechanism of DOX cardiotoxicity.

Mitochondria have been identified as one of the major targets in DOX-induced subcellular damage in the heart.^24, 25^ Mitochondrial dysfunction is related to cardiotoxicity. Preservation of mitochondrial integrity is essential for maintaining energy production and preventing cell death. Mitochondria constantly undergo fusion and fission, which are necessary for the maintenance of organelle fidelity.^15, 26^ Abnormal mitochondrial fission is involved in the initiation of apoptosis.^27^ Various proteins participate in the regulation of mitochondrial dynamics. Mitofusin 1 (Mfn1), Mfn2 and optic atrophy 1 (OPA1) promote mitochondrial fusion and inhibit apoptosis.^28, 29^ Drp1, fission 1 (Fis1) and mitochondrial fission factor (Mff) induce mitochondrial fission and apoptosis.^30, 31, 32^ Accumulating evidence show that abnormality in mitochondrial dynamics can provoke the occurrence of heart disease.^26^ Our previous studies have demonstrated that mitochondrial dynamic regulators such as Drp1, Fis1 and Mff participate in cardiomyocyte apoptosis induced by hypoxia or H_2_O_2_.^33, 34, 35^ In this study, we first report the role of mitochondrial fission in the DOX cardiotoxicity, and we find that Drp1-mediated mitochondrial fission is required for DOX cardiotoxicity. It would be interesting to elucidate the relationship between mitochondrial fission and other events such as ROS generation in the apoptotic cascades of DOX. It remains to be further elucidated whether Mfn1, Mfn2, Mff and Fis1 are involved in regulating cardiotoxicity.

ARC is evolutionarily expressed at a high level in the heart and has an important role in cardioprotection through antiapoptosis. It was originally identified to be a caspase-inhibiting protein and can specifically inhibit the activation of caspase-2 and -8, thereby blocking apoptosis induced by a variety of stimuli.^6^ Further studies revealed that ARC may also elicit its antiapoptotic function by other means. It can interact with Fas, FADD and Bax,^8, 36^ inhibit Cyt *C* release^37^ and maintain *ΔΨ*m.^7^ Our present study reveals that ARC can inhibit DOX-induced mitochondrial fission in cardiomyocytes. It is possible that ARC elicits its effects against the collapse of *ΔΨ*m and Cyt *C* release through inhibiting mitochondrial fission. We and others have previously shown that dysregulation of mitochondrial dynamics was involved in the heart under pathological conditions, such as oxidative stress and hypoxia.^33, 34, 38^ ARC is downregulated under these pathological conditions in the heart.^9^ It is remains to be further elucidated whether ARC is involved in the maintenance of mitochondrial homeostasis under these pathological conditions.

The molecular mechanism by which ARC inhibits mitochondrial fission is not fully understood. Our previous study has shown that ARC can inhibit the recruitment of Drp1 from the cytosol to the mitochondrial surface.^13^ But the detailed mechanisms remain need to be further elucidated. ARC is predominantly distributed in the mitochondria in cardiomyocytes.^39^ Mitochondrial dynamic regulators such as Mfn1, Mfn2, Fis1, Mff and OPA1 are also localized to mitochondria.^29, 30, 31, 40^ Drp1 is mainly located in the cytosol and can be recruited to the outer membrane of mitochondria during mitochondrial fission.^41^ The relationship between ARC and other fusion/fission factors in the apoptotic pathway of cardiomyocytes needs to be determined in the future study.

MiRNAs have an important role in cardiac development and function maintenance. Aberrant expression of miRNAs in the heart is related to heart disease.^42, 43^ It has been shown that upregulation of miR-146a after DOX treatment is involved in DOX cardiotoxicity by targeting ErbB4.^44^ Upregulation of miR-1 and miR-133 contributes to arsenic-induced cardiac electrical disorders.^45^ Only a few miRNAs have been found to be involved in cardiotoxicity. Other miRNAs that participate in cardiotoxicity need to be further explored in the future study. The present study demonstrates that miR-532-3p participates in apoptosis and cardiotoxicity induced by DOX. A variety of stimuli can trigger apoptosis in cardiomyocytes, including Fas ligand, anoxia, serum deprivation, mechanical stretch and oxidative stress.^9, 46, 47, 48^ It remains to be determined as to whether miR-532-3p is involved in apoptosis induced by these stimuli. Also whether miR-532-3p is involved in pathological insults to the hearts is an interesting question for future investigation.

DOX-induced cardiotoxicity is involved in a complex molecular process. Our present work has identified that miR-532-3p contributes to the downregualtion of ARC. Our results suggested that enforced expression of ARC or knockdown miR-532-3p cannot completely reverse the DOX effect. Our finding is only one point of the complicated cascades, and our finding does not exclude the involvements of any other molecules and/or pathways that constitute to the complex machinery. It would be interesting to explore how miR-532-3p–ARC pathway is involved in this complex molecular process in the future study.

Recent studies showed that ARC is highly expressed in many malignant tumors.^13, 23^ Our previous work has proved that highly expressed ARC contributed to cancer cells resistance to chemotherapy by targeting the mitochondrial fission machinery. It was also demonstrated that ARC expression was downregulated in cancer cells following DOX treatment. Downregulation of ARC can sensitize cancer cells to DOX-induced apoptosis and chemotherapy. The inducement of apoptosis is beneficial for the treatment of cancers. In contrast, heart is an organ composed of terminally differentiated postmitotic cardiac myocytes. Inducement of apoptosis in cardiomyocytes may lead to cardiotoxicity and pathological disorders. The cardiotoxicity induced by DOX limited its usefulness in chemotherapy. In light of the key role of ARC in controlling DOX-induced apoptosis both in cardiomyocytes and cancer cells, it is necessary to find out the molecular approaches that can downregulate ARC expression in cancer cells but upregulated ARC in cardiomyocytes. In this study, we reveal that miR-532-3p is upregulated in cardiomyocytes administered with DOX. MiR-532-3p sensitizes cardiomyocytes to apoptosis by negatively regulating ARC. But, in cancer cells, the expression levels of miR-532-3p are low compared with cardiomyocytes and do not change in response to DOX. DOX-induced apoptosis was attenuated by miR-532-3p antagomir in cardiomyocytes but not in cancer cells. These results suggest that inhibition of miR-532-3p may be a novel strategy to conquer cardiotoxicity and not disturb chemotherapeutic effect for cancer patients.

Taken together, we report here that the upregulated expression of miR-532-3p in cardiomyocytes exposed to DOX *in vitro* and *in vivo* is involved in DOX cardiotoxicity by targeting ARC. There is emerging evidence for the involvement of ARC in cardioprotection. Therefore, the development of new therapeutic strategies based on ARC, such as delivery of nucleotides that inhibit miR-532-3p, is promising for overcoming the cardiotoxicity of chemotherapy for cancer therapy.

## Materials and Methods

### Cell culture and treatment

Neonatal rat and mouse cardiomyocytes were isolated and cultured as we described elsewhere.^10^ For detailed methods, please refer to Supplementary Information. Hela cells and human gastric cancer cell line SGC-7901 were as we described elsewhere.^13^ Human hepatocellular carcinoma cell line HepG-2 and human colorectal cancer cell line SW-480 were purchased from Chinese Academy of Sciences Cell Bank. The cells were cultured in Dulbecco's modified Eagle's medium (GIBCO, Grand Island, NY, USA) supplemented with 10% fetal bovine serum, 100 units/ml penicillin and 100 *μ*g/ml streptomycin in a humidified atmosphere containing 5% CO_2_ at 37 °C. The treatment with DOX (Sigma, St. Louis, MO, USA) was performed as we described elsewhere.^13^

### Cell death assay and caspase-3 activity assay

Cell death was determined by Trypan Blue Exclusion. The Trypan Blue-positive and Trypan Blue-negative cells were counted on a hemocytometer (Shanghai Anxin Optical Instrument Manufacture Co. Ltd, Shanghai, China). Apoptosis was measured by the Alexa Fluor 488 annexin V/Dead Cell Apoptosis Kit (Invitrogen, Carlsbad, CA, USA) and was performed on a FACS Calibur (Becton Dickinson, Franklin Lakes, NJ, USA). All Annexin V-positive cells (Annexin V+) were considered as apoptotic cells. Caspase-3 activity was measured using an Apo-ONE Homogeneous Caspase-3/7 Assay Kit (Promega, Madision, WI, USA) according to the manufacturer's protocol.

### Adenovirus and infection

Adenovirus ARC (AdARC) and adenovirus *β*-galactosidase (Ad*β*-gal) were prepared as we described elsewhere.^10^ The adenoviruses harboring RNAi sequence were constructed using pSilence Adeno 1.0-CMV system (Ambion, Grand Island, NY, USA) as we described elsewhere.^12^ The rat ARC RNAi target sequence is 5′-ACTGTGAGCATGCCAGACC-3′ and the scrambled RNAi sequence is 5′-GTGCATCAGACTACCAGGC-3′. The rat Drp1 RNAi target sequence is 5′-CTGGAGAGGAATGCTGAAA-3′ and the scramble RNAi sequence is 5′-CTGGAAATGGAGGGAACTA-3′. All adenoviruses were amplified in HEK-293 cells. Adenoviral infection of cells was performed as we described previously.^13^

### Cell transfection with miRNA duplexes or antagomirs

The miR-532-3p duplexes were synthesized by GenePharma Co. Ltd (Shanghai, China). MiR-532-3p mimic sequence was 5′- CCUCCCACACCCAAGGCUUGCA-3′. Mimic control sequence was 5′-UUCUCCGAACGUGUCACGUTT-3′. Chemically modified antisense oligonucleotides (antagomirs) were used to inhibit endogenous miR-532-3p expression. The antagomir sequence was 5′-UGCAAGCCUUGGGUGUGGGAGG-3′. All the bases were 2′-O-methyl-modified (GenePharma Co. Ltd). The antagomir control sequence was 5′-CAGUACUUUUGUGUAGUACAA-3′. All the bases were 2′-O-methyl-modified (GenePharma Co. Ltd). Cells were transfected with miRNA duplexes (100 nM) or antagomirs (100 nM) using Lipofectamine 2000 (Invitrogen, Grand Island, NY, USA) according to the manufacturer′s instructions.

### Immunoblotting

Immunoblotting was performed to determine the expression levels of ARC, Drp1 and Cyt *C* as we described previously.^13^ For details, please refer to Supplementary Information. Anti-ARC antibody was obtained from Abcam (Cambridge, UK). Anti-Drp1 antibody and anti-Cyt *C* antibody were obtained from Becton Dickinson.

### Quantitative real-time PCR

qRT-PCR was carried out to evaluate the expression level of miRNAs and ARC mRNA. For details, please refer to Supplementary Information.

### Mitochondrial staining

We carried out mitochondrial staining as described.^33^ Briefly, we plated the cells onto the coverslips. After treatment, we stained them for 20 min with 0.02 *μ*M MitoTracker Red CMXRos (Molecular probes, Inc., Eugene, OR, USA). We imaged mitochondria by using a laser scanning confocal microscope (Zeiss LSM510 META, Jena, Germany). For each experiment, we randomly measured at least 150 cells to determine the percentage of cells with mitochondrial fission.

### Electron microscopy

Heart ultrastructural analysis was performed to quantify mitochondrial fission as we described elsewhere.^33^ For details, please refer to Supplementary Information.

### Animal experiments

ARC transgenic mice were generated as we described previously.^12^ Adult ARC transgenic mice and their wild-type littermates (C57BL/6, male, 10 weeks old) were injected with DOX or saline as previously reported.^4^ Briefly, mice were treated once a week with 4 mg/kg DOX or saline solution control for 4 weeks. Heart tissues were analyzed 1 week after the last treatment. Cardiac function and left ventricular remodeling were investigated 2 weeks after the last treatment. All animal experiments were performed according to the protocols approved by the Animal Care Committee, Institute of Zoology, Chinese academy of Sciences, Beijing, China. For detailed information on echocardiographic assessment and histology observation, please refer to Supplementary Information.

### Statistical analysis

All statistical analyses were performed using the SPSS 13.0 statistical software (SPSS Inc., Chicago, IL, USA). The results are expressed as means±S.D. of at least three independent experiments. Paired data were evaluated by Student's *t*-test. We used a one-way ANOVA for multiple comparisons. A value of *P*<0.05 was considered significant.

Additional Materials and Methods are available in Supplementary Information.

## Abbreviations

DOX: doxorubicin
ARC: apoptosis repressor with caspase recruitment domain
miR-532-3p: microRNA-532-3p
Mfn1: mitofusin 1
OPA1: optic atrophy 1
Drp1: dynamin-related protein 1
Fis1: fission 1
Mff: mitochondrial fission factor
*ΔΨ*m: mitochondrial membrane potential
Cyt *C*: cytochrome *C*
