# Supplementary material for: MicroRNA-532-3p regulates mitochondrial fission through targeting apoptosis repressor with caspase recruitment domain in doxorubicin cardiotoxicity
Source: Cell Death Dis. 2015 Mar 12;6(3):e1677–. doi: 10.1038/cddis.2015.41 (PMC4385919; doi:10.1038/cddis.2015.41)
Supplement: Supplementary Materials and Methods [file cddis201541x1.pdf]

Supplemental Materials and Methods

Isolation and culture of Neonatal rat cardiomyocytes and mouse cardiomyocytes.

Neonatal rat cardiomyocytes were isolated form 2-day-old Wistar rats. In brief, after dissection hearts were washed, minced in HEPES-buffered saline solution contained: 130 mM NaCl, 3 mM KCl, 1 mM NaH_2PO_4, 4 mM glucose and 20 mM 4-(2-hydroxyethyl)-1-piperazineethanesulfonic acid (HEPES) (pH adjusted to 7.35 with NaOH). We dispersed the tissues in a series of incubations at 37 ℃ in HEPES-buffered saline solution containing 1.2 mg/ml pancreatin and 0.14 mg/ml collagenase (Worthington). After centrifugation cells were re-suspended in Dulbecco's modified Eagle medium/F-12 (GIBCO) containing 5% heat-inactivated horse serum, 0.1 mM ascorbate, insulin-transferring-sodium selenite media supplement, 100 U/ml penicillin, 100 μg/ml streptomycin, and 0.1 mM bromodeoxyuridine. We pre-plated the dissociated cells at 37 ℃ for 1 h, diluted them to 1×10^6 cells/ml and plated them in 10 μg/ml laminin-coated different culture dishes according to the specific experimental requirements.

Primary neonatal mouse cardiomyocytes were isolated from hearts of 1-day-old ARC transgenic mice (Tg) and wide type mice (WT), as we described in isolation of neonatal rat cardiomyocytes with minor modifications. Briefly, non-myocyte contaminants were removed by two rounds of pre-plating for 1.5 h on 100-mm plastic cell culture dishes in a humidified incubator at 37 °C with 5% CO2. Cardiomyocytes were plated into laminin-coated different culture dishes with serum-containing medium. Following a 24-h incubation period, serum-containing medium was replaced

with no serum medium.

Immunoblotting.

Immunoblotting was performed to determinate the expression level of ARC, Drp1 and Cyt C. Briefly, cells were lysed for 1 h at 4℃ in a lysis buffer (20 mM Tris pH 7.5, 2 mM EDTA, 3 mM EGTA, 2 mM DTT, 250 mM sucrose, 0.1 mM phenylmethylsulfonyl fluoride, 1% Triton X-100) containing a protease inhibitor cocktail. Protein samples were subjected to 12% SDS-PAGE and transferred to nitrocellulose membranes. Blots were probed using corresponding primary antibodies. Then the horseradish peroxidase-conjugated secondary antibodies were used. Antigen-antibody complexes were tested by enhanced chemiluminescence.

Mitochondrial membrane potential (ΔΨm) detection.

For the detection of ΔΨm, 100nM tetramethylrhodamine ethyl ester (TMER) (Molecular Probes, Invitrogen) was added, and the mixture was incubated for 15min at 37℃. The red TMRE fluorescence was detected by confocal microscopy and the fluorescence intensity was measured by the 543/590 excitation/emission filter set.

Quantitative real-time PCR (qRT-PCR).

Stem-loop qRT-PCR was carried out on an Applied Biosystems ABI Prism 7000 sequence detection system. Total RNA was extracted using Trizol reagent. After DNase I (Takara, Otsu, Japan) treatment, RNA was reverse-transcribed with reverse transcriptase kit (Takara). Mature miR-532-3p, miR-664, miR-24-1-3p, miR-134-5p, miR-150 and miR-28-5p levels were measured using SYBR Green Realtime PCR Master Mix (Takara) according to the manufacturer’s instructions. The reverse primer

for all miRNAs was same and the sequence was 5'-GTGCAGGGTCCGAGGT-3'. The sequence of miR-532-3p forward primer was 5'-ATCCTCCCACACCCAAGG -3'. The sequence of miR-664 forward primer was 5'-CGCCGTATTCATTTACTCCCC -3'. The sequence of miR-24-1-3p forward primer was 5'-CGCCTGGCTCAGTTCAGCAG -3'. The sequence of miR-134-5p forward primer was 5'- GCCTGTGACTGGTTGACCA -3'. The sequence of miR-150 forward primer was 5'- GCCTCTCCCAACCCTTGTA -3'. The sequence of miR-28-5p forward primer was 5'- CCCAAGGAGCTCACAGTCT -3'. The levels of these miRNAs analyzed by qRT-PCR were normalized to that of U6. The sequences of U6 primers were: forward, 5'-GCTTCGGCAGCACATATACTAA-3'; reverse, 5'-AACGCTTCACGAATTTGCGT-3'. Quantitative detection of ARC, ANP and β-MHC was performed using the same strategy. The primers used for ARC were: forward, 5'-ATGGGTAACATGCAGGAGCGC-3'; reverse, 5'-GTCCAGCAGCAACCCAGAGTC -3'. The sequence of ANP primers were forward: 5'-CTCCGATAGATCTGCCCTCTTGAA-3' and reverse: 5'-GGTACCGGAAGCTGTTGCAGCCTA-3'. β-MHC forward primer was 5'-CAGACATAGAGACCTACCTTC-3' and reverse was 5'-CAGCATGTCTAGAAGCTCAGG-3'. The mRNAs levels were normalized to that of glyceraldehyde-3-phosphate dehydrogenase (GAPDH). The sequences of GAPDH primers were: forward, 5'- TGGAGTCTACTGGCGTCTT -3'; reverse, 5'-TGTCATATTTCTCGTGGTTCA -3'.

Electron microscopy.

Heart ultrastructural analysis was performed to quantify mitochondrial fission as we described [1]. Briefly, we carried out sample preparations and conventional EM as described before [2]. Samples were examined at a magnification of 15,000 with a JEOL JEM-1230 transmission electron microscope. For comparison of mitochondrial fission, we evaluated EM micrographs of thin sections. We measured the size of individual mitochondrion by using Image Pro Plus software. For each experiment, we measured approximately 100 mitochondria in a representative area to determine the percentage distribution of mitochondria with various sizes. In DOX-treated heart tissues, mitochondria disintegrated into numerous small round fragments of varying size, the number of small mitochondrion was increased. Thus, we determined the mitochondria smaller than 0.6 μm^2 to be fission mitochondria.

Echocardiographic assessment.

Echocardiography was performed as we have described.[1] Generally, the mice were mildly anesthetized and transthoracic echocardiography was performed using a Vevo 770 high-resolution system (Visualsonics, Toronto, Canada). Two-dimensional guided M-mode tracings were recorded in both parasternal long and short axis views at the level of papillary muscles. Systolic left-ventricular internal diameter (LVIDs) and diastolic left-ventricular internal diameter (LVIDd) were measured. We calculated fractional shortening (FS) of left ventricular diameter as [(LVIDd – LVIDs)/LVIDd] × 100. All of the measurements were made from more than three beats and averaged. After *in vivo* evaluation of cardiac function we euthanized the mice, harvested and weighted the hearts and used them for histological examination.

Histology.

We fixed the harvested hearts in 10% formalin, embedded them in paraffin and sectioned them at 6 μm thickness. We performed TUNEL staining according to manufacturer's instructions (Roche). We stained the cardiomyocytes with antibody to α-actinin (A7811, Sigma) and the total nuclei with 4′,6-diamidino-2-phenylindole (DAPI). An investigator blind to the treatment quantified 10 random fields of samples. To determine LV remodeling after DOX injury, we stained the heart sections with standard Masson trichrome staining (Sigma) and Rhodamine-conjugated wheat germ agglutinin (Sigma) as described.[1]

Reporter constructions and luciferase assay.

The fragment of ARC 3'UTR containing miR-532-3p binding site was amplified from rat genomic DNA by PCR. The forward primer was 5'-GAGAACGAATTCACCCTAGCTTGCAGGCTT-3', the reverse primer was 5'-CCATCTACTAGTGCTTGGAAACACAGCTGG-3'. To generate reporter vector containing miR-532-3p binding sites, the PCR product was cloned downstream of the stop codon of the luciferase gene of pGL3 vector (Promega, Madison, WI, USA). To generate ARC 3′UTR-Mut, the mutation (the wild-type ARC 3′UTR Site: CUGGGAGC, ARC 3'UTR-Mut: CUAAACGC) were produced using the QuikChange II XL Site-Directed Mutagenesis Kit (Stratagene, La Jolla, CA, USA). For luciferase assay performed in HEK-293, cells in 24-well plates were co-transfected with 200 ng per well luciferase reporter constructs, 400 ng per well miR-532-3p mimic or mimic control using Lipofectamine 2000 (Invitrogen). 5 ng per

well SV-Renilla luciferase plasmids served as the internal control. Cells were harvested at 24 h after transfection and the luciferase activity was detected using the Dual Luciferase Reporter Assay kit (Promega) according to the manufacturer's instructions. 30μl protein samples were analyzed in a luminometer. Firefly luciferase activities were normalized to Renilla luciferase activity. The similar strategy was used to perform luciferase analysis in cardiomyocytes.

Reference

1. Wang JX, Jiao JQ, Li Q, Long B, Wang K, Liu JP*, et al.* miR-499 regulates mitochondrial dynamics by targeting calcineurin and dynamin-related protein-1. *Nature medicine* 2011, **17**(1)**:** 71-78.

2. Elrod JW, Calvert JW, Morrison J, Doeller JE, Kraus DW, Tao L*, et al.* Hydrogen sulfide attenuates myocardial ischemia-reperfusion injury by preservation of mitochondrial function. *Proc Natl Acad Sci U S A* 2007, **104**(39)**:** 15560-15565.

Supplemental Figure Legends

Supplemental Figure 1 DOX induces mitochondrial fission and cell death.

(**a**) Mitochondria were stained by Mitotracker red in neonatal rat cardiomyocytes exposed to 2μM DOX for indicated times. The percentage of cells undergoing mitochondrial fission was counted and summarized. (**b**) Cell death was analyzed by Trypan Blue Exclusion from cardiomyocytes treated as described in A. (**c**) Caspase-3 activition was analyzed in neonatal rat cardiomyocytes treated as described in A. *$p<0.05$ versus control (untreated). (**d** and **e**) Apoptosis was analyzed by Annexin V/PI assay. Neonatal rat cardiomyocytes were infceted with ARC (d) or ARC siRNA (e) and then treated with 2μM DOX (d) or 0.2μM DOX (e) for 12 hours. Representative images showed apoptosis (Annexin V-positive, left) and the quantitative analysis of apoptosis was shown (right). Data represent mean ± SD, n=3. *$p<0.05$ versus DOX alone.

Supplemental Figure 2 Mitochondrial fission is involved in the initiation of apoptosis induced by DOX.

(**a**-**c**) Knockdown of Drp1 using its small interfering RNA (siRNA) (**a**) reduced mitochondrial fission (**b**) and cell death (**c**) upon DOX (2μM) treatment. (**d-f**) Enforced expression of Drp1 by transfecting with Drp1 (**d**) sensitized DOX to induce mitochondrial fission (**e**) and cell death (**f**). (**g**) The release of Cyt C was measured in neonatal rat cardiomyocytes treated as decribed in B. Cardiomyocytes were then fractionated to collect the cytosolic fraction for immunoblot analysis of Cyt C. (**h**) The mitochondrial menbrabe potential ($\Delta\Psi$) was determined by the fluorescence

intensity of TMRE in cardiomyocytes treated as decribed in B. Data shown are mean ± SD, n=3. *p<0.05.

Supplemental Figure 3 ARC was downregulated in mice administrated with DOX. (**a** and **b**) ARC protein levels (**a**) and mRNA levels (**b**) in mice administrated with DOX or saline. Data are presented as mean ± SD, *n*=5 mice per group. *p<0.05.

S**upplemental Figure 4** ARC is regulated by miR-532-3p.

(**a**) MiRNAs levels in cardiomyocytes treated with DOX. Neonatal rat cardiomyocytes were treated with 2μM DOX for 24 hours, the expression levels of miR-532-3p, miR-664, miR-24-1-3p, miR-134-5p, miR-150 and miR-28-5p were detected by qRT-PCR. *p<0.05 versus control (untreated). (**b**) MiR-532-3p levels in cardiomyocytes transfected with miR-532-3p mimic or negative control. (**c**) MiR-532-3p levels in cardiomyocytes transfected with miR-532-3p antagomir or antagomir control and treated with 2μM DOX for 12 hours. (**d**) Schematic diagram of the reporter constructs containing putative miR-532-3p binding site in ARC 3′UTR, mutations introduced into miR-532-3p binding site in rat ARC 3’UTR were labeled in Red. (**e**) ARC mRNA levels in ARC transgenic mice (Tg) cardiomyocytes transfected with miR-532-3p or negative control. Neonatal mouse cardiomyocytes were isolated from ARC Tg mice or WT mice as described in supplemental methods. Data represent mean ± SD, n=3. *p<0.05

Supplemental Figure 5 MiR-532-3p is not involved in DOX induced apoptosis in cancer cells.

(**a** and **b**) The miR-532-3p levels in SW-480 (**a**) and HepG-2 (**b**) cells treated with 2μM DOX for indicated times. (**c** and **d**) Cell death in SW-480 (**c**) and HepG2 (**d**) cells transfected with miR-532-3p antagomir and treated with 2μM DOX. Data represent mean ± SD, n=3.
