# Supplementary material for: MicroRNA-532-3p regulates mitochondrial fission through targeting apoptosis repressor with caspase recruitment domain in doxorubicin cardiotoxicity
Source: Cell Death Dis. 2015 Mar 12;6(3):e1677–. doi: 10.1038/cddis.2015.41 (PMC4385919; doi:10.1038/cddis.2015.41)

Supplemental Figure 1

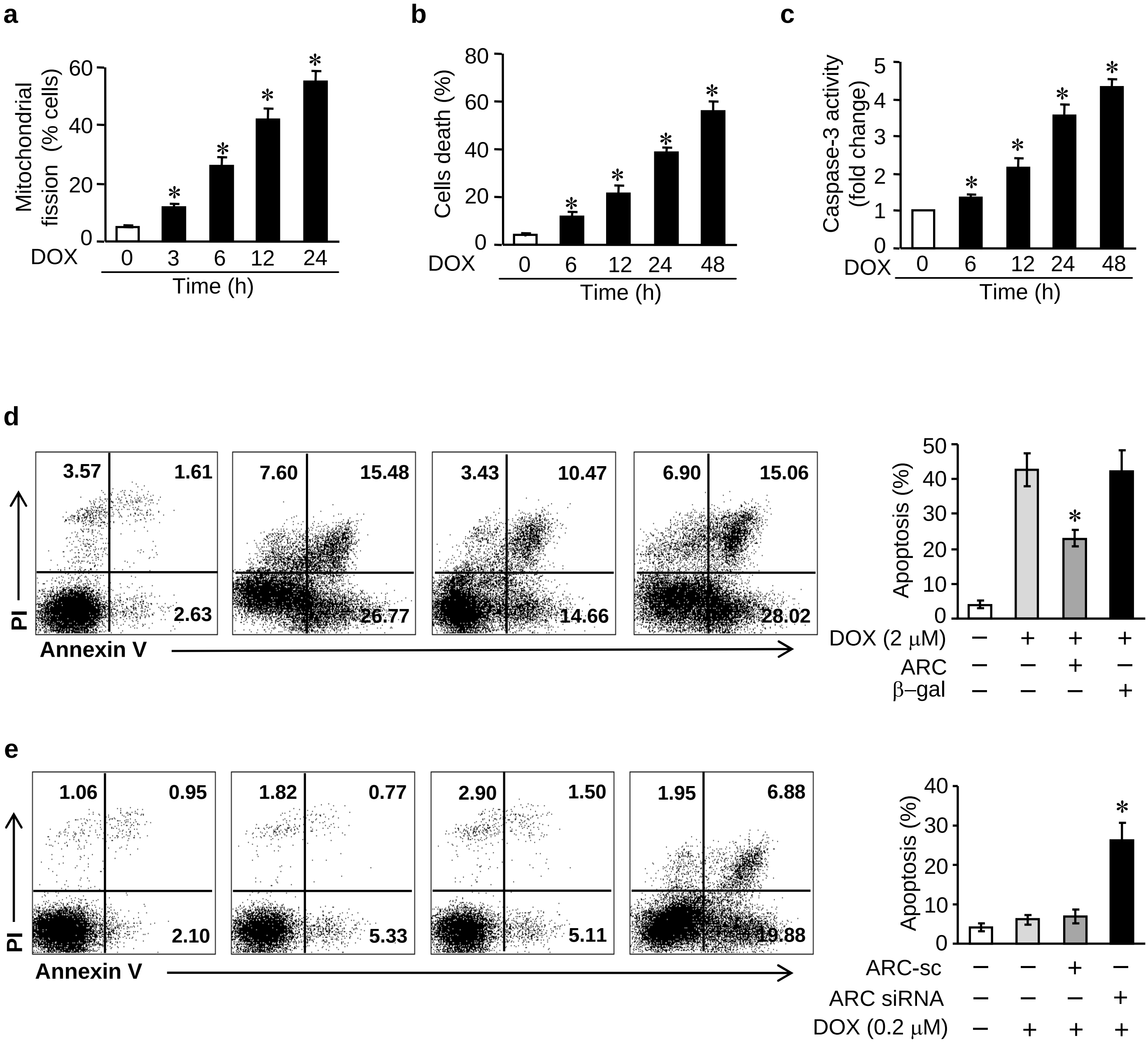

Supplemental Figure 2

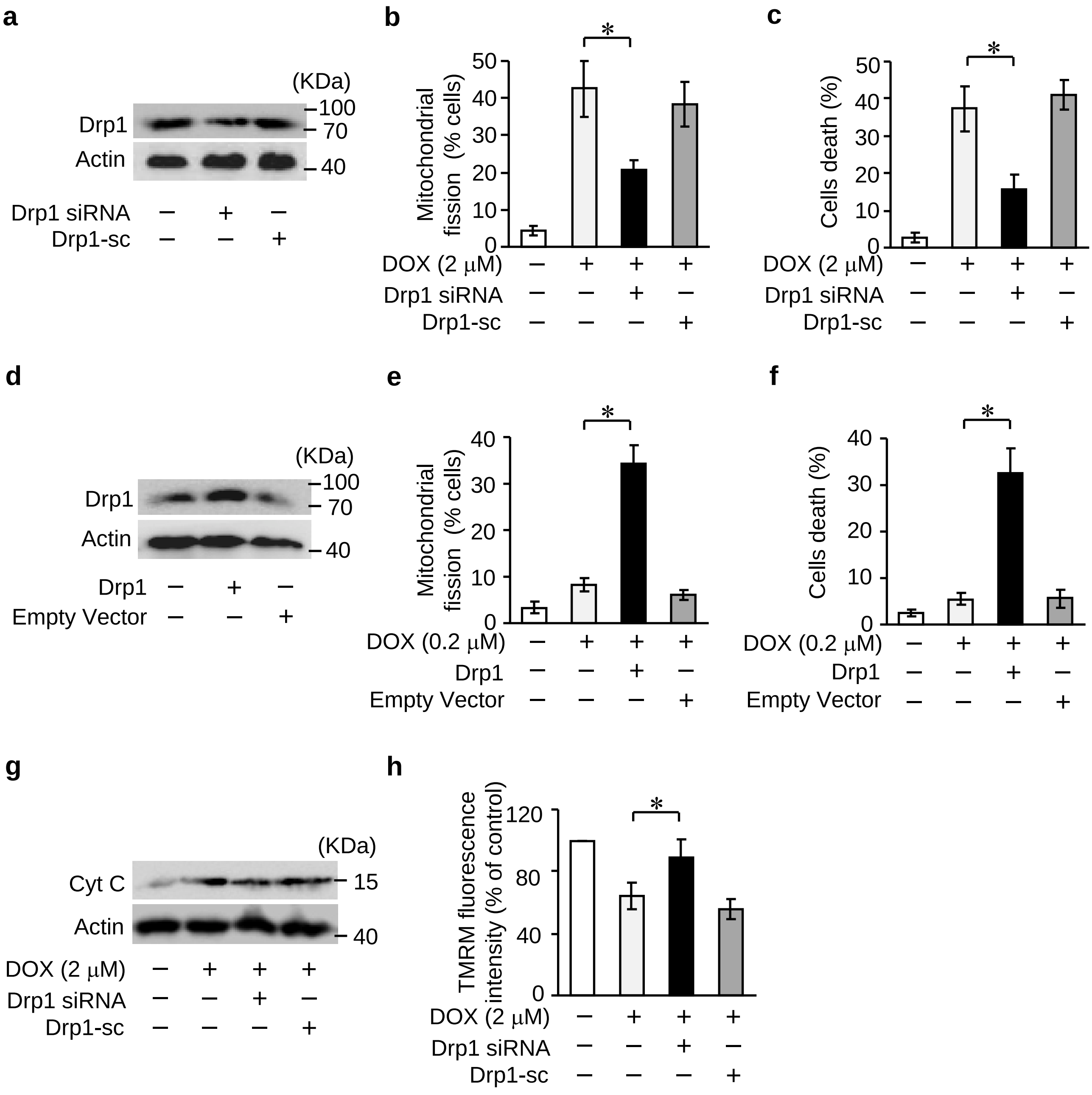

Supplemental Figure 3

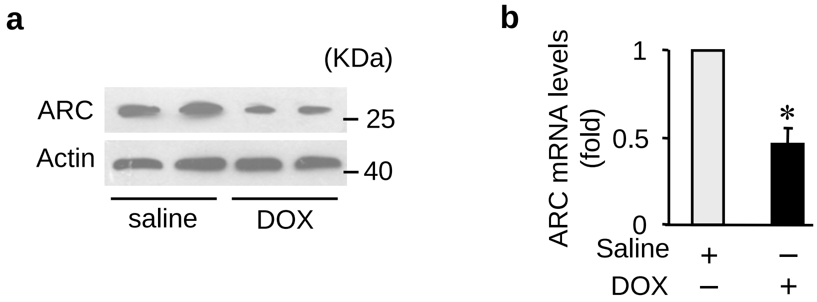

Supplemental Figure 4

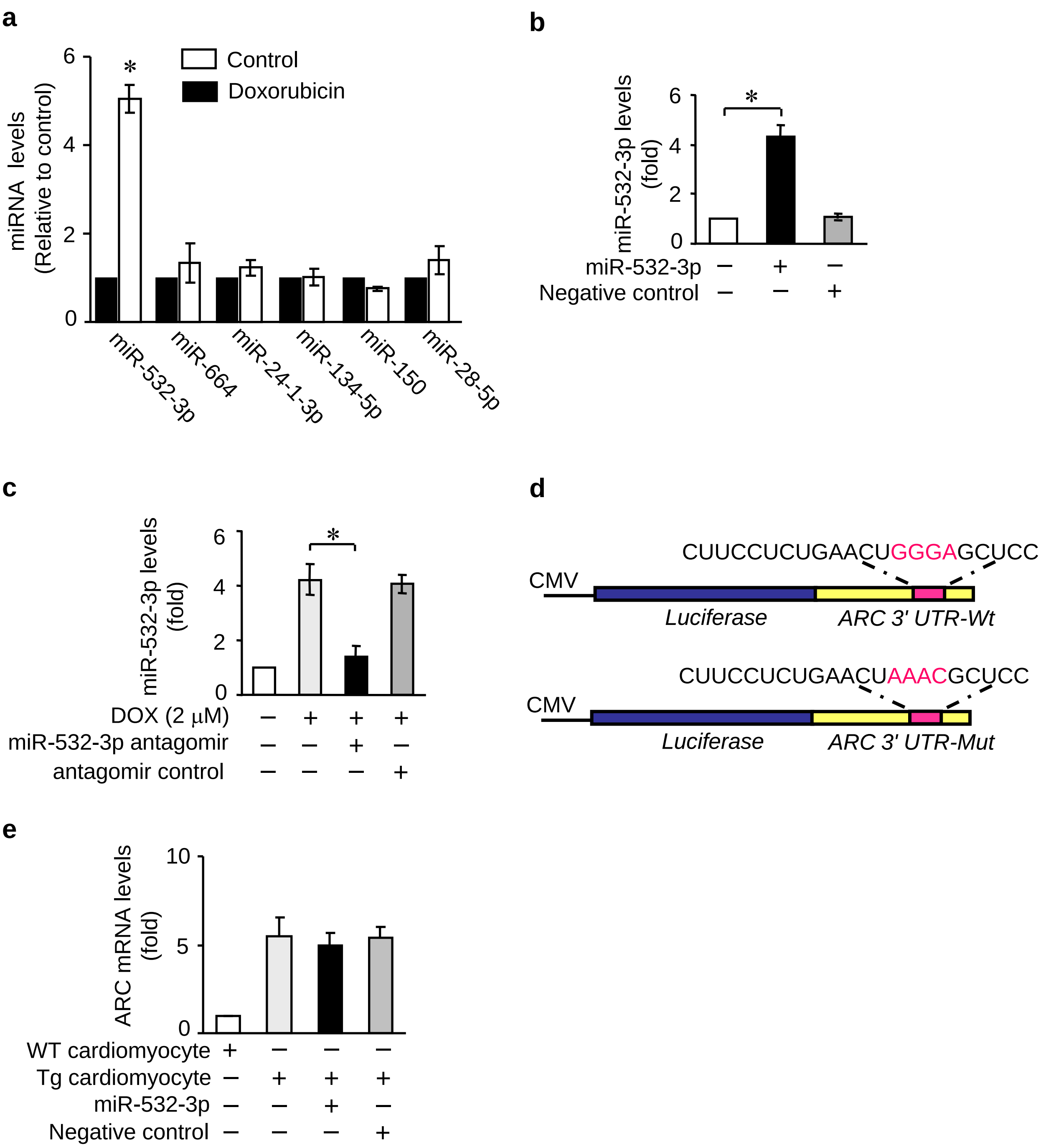

Supplemental Figure 5

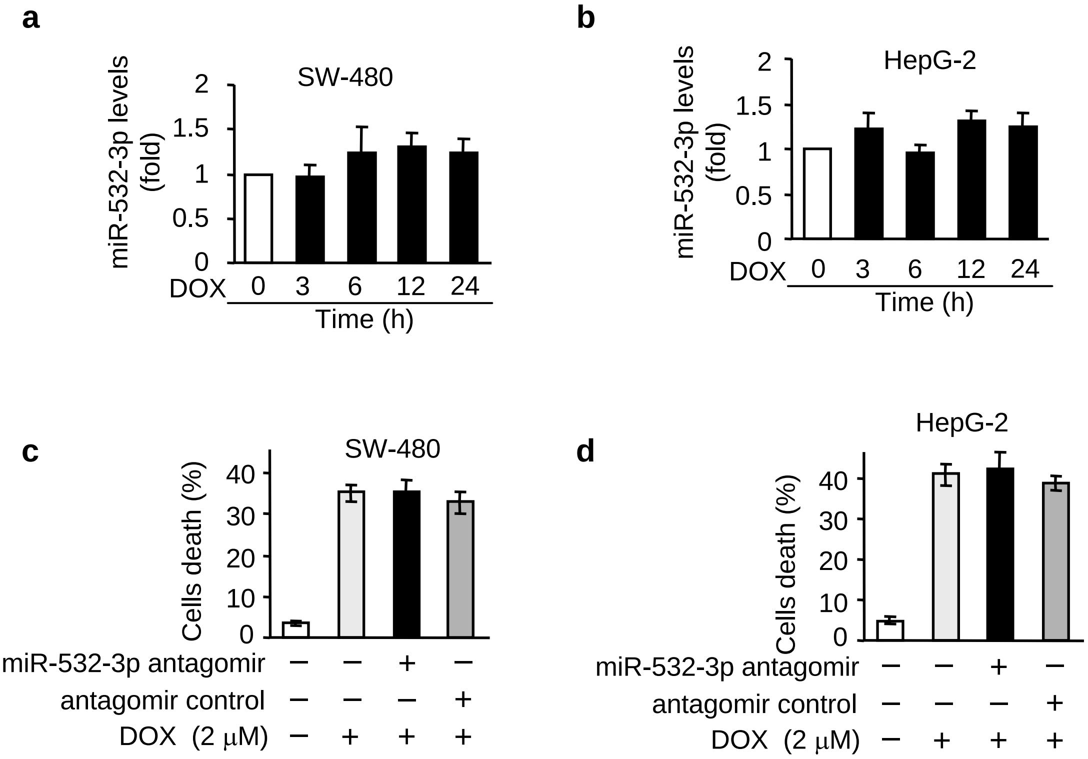

Supplement: Supplementary Figures [file cddis201541x2.pdf]
